# Needs analysis of learning objectives for a lifestyle medicine curriculum in family medicine residency program: an E-Delphi study

**DOI:** 10.1186/s12875-026-03421-3

**Published:** 2026-06-16

**Authors:** Vildan Mevsim, Yasemin Özkaya, Duygu Ayhan Başer, Gökçe İşcan, Merve Saniye İmançer, Oğulcan Çöme, Tolga Günvar, Irfan Yurdabakan

**Affiliations:** 1 Dokuz Eylul University Faculty of Medicine Department of Family Medicine, Izmir, Turkey; 2 Department of Family Medicine, Izmir City Hospital, Izmir, Turkey; 3 Department of Family Medicine, Hacettepe University Faculty of Medicine, Ankara, Turkey; 4 Department of Family Medicine, Suleyman Demirel University Faculty of Medicine, Isparta, Turkey; 5 Department of Educational Measurement and Evaluation, Dokuz Eylül University Faculty of Education, Izmir, Turkey

**Keywords:** Lifestyle medicine, Family medicine, Core competencies, E-Delphi, Curriculum development

## Abstract

**Background:**

Lifestyle Medicine (LM) plays a critical role in the prevention, treatment, and even reversal of chronic diseases through evidence-based lifestyle interventions. Despite its importance, LM is not yet systematically embedded into family medicine residency training. Current approaches often focus narrowly on behavior change and lack an integrated curriculum framework. This study aimed to identify and reach expert consensus on the core learning objectives necessary for incorporating LM into family medicine specialty education in Turkey.

**Methods:**

A two-round electronic modified Delphi (E-Delphi) method was employed to gather consensus from a multidisciplinary expert panel. Fifteen participants—including family medicine academicians, practicing physicians, residents, and professionals in medical education, nutrition, and sports medicine—evaluated 114 learning objectives developed by a steering committee through literature review. A 5-point Likert scale was used for scoring, with consensus defined as ≥ 80% agreement, a median score ≥ 4, and an interquartile range (IQR) ≤ 1.

**Results:**

In the first round, consensus was achieved for 107 objectives. In the second round, 96 learning objectives reached final consensus, covering eight core domains: introduction to LM, nutrition, physical activity, sleep health, stress management, substance use, sexual health, social relationships, and environmental exposures. Highest agreement was observed in nutrition and physical activity; objectives related to stress regulation, sleep assessment, and environmental policy received lower consensus, indicating areas needing further curricular clarification.

**Conclusion:**

The findings provide a foundational framework for curriculum development and may guide international efforts to integrate LM into postgraduate medical education.

## Introduction

Lifestyle refers to the daily habits and behaviors that shape how individuals live and play a key role in health, well-being, and disease prevention [1].

Lifestyle Medicine (LM) refers to the non-pharmacological and non-surgical strategies employed in the management of chronic diseases. Its conceptual origins can be traced back to the preventive medicine framework of the 1960s, which emphasized lifestyle modification, public health screenings, safety measures, and immunization programs as central elements of disease prevention [2]. However, the structured and contemporary form of LM began to take shape following the epidemiological shift of the 1980s, a period during which chronic diseases supplanted infectious diseases as the leading health burden. This transition highlighted the significant impact of lifestyle factors such as tobacco use, unhealthy diet, and physical inactivity on public health. Consequently, the term “Lifestyle Medicine” gained prominence in both clinical and academic contexts [3].

The term LM was first officially defined by Dr. James Rippe in 1999, with the publication of the inaugural edition of his book, Lifestyle Medicine [4]. According to Rippe, LM is “an evidence-based discipline that supports individuals and communities in addressing the underlying causes of chronic diseases by implementing comprehensive lifestyle changes—including nutrition, physical activity, stress management, social support, and reducing environmental exposures—to prevent, manage, and even reverse disease progression” [5].

Although primary care is ideally suited to provide holistic care beyond symptom management [6], this potential is not consistently realized in practice. Lifestyle-based interventions including dietary modification, increased physical activity and weight management have demonstrated efficacy not only in preventing but also in reversing conditions such as type 2 diabetes and early-stage prostate cancer [7, 8]. Moreover, they are associated with reduced reliance on pharmacological treatments and improvements in quality of life [9].

In Türkiye, primary care is delivered through a family medicine system in which family physicians serve as the first point of contact and provide comprehensive, continuous, and preventive care to a registered population. Lifestyle-related services are primarily delivered within Family Health Centers (FHCs), where physicians are responsible for preventive counselling, chronic disease management, and health promotion activities. In recent years, additional institutional structures such as Healthy Life Centers have been introduced to support lifestyle medicine practices. These centers offer multidisciplinary services, including nutrition counselling, physical activity guidance, smoking cessation support, and psychosocial interventions, often involving professionals such as dietitians, psychologists, and physiotherapists [10]. While pharmacological treatment remains a component of chronic disease management in primary care, there has been an increasing emphasis on non-pharmacological, lifestyle-based interventions. However, the integration of these services into routine practice and training remains variable, highlighting the need for structured educational frameworks in lifestyle medicine within family medicine training.

With 71% of global deaths attributable to chronic conditions linked to modifiable behavioural factors [11]. Integrating lifestyle medicine into routine clinical practice has the potential to be cost-effective and provide long-term benefits [5], while also empowering patients to actively manage their health, which may improve clinical outcomes and patient satisfaction [12, 13]. However, further large-scale studies across different primary care systems are needed to establish its cost-effectiveness more conclusively.

Despite growing recognition of its value, many healthcare providers receive limited or no structured training in lifestyle medicine, encompassing both foundational knowledge (e.g., nutrition, physical activity, and behavioral science) and practical counselling skills, which limits its effective integration into clinical practice [1]. While elements of lifestyle change are present in family medicine residency programs, LM as a discipline extends beyond individual behaviour modification to encompass broader and more complex determinants. As a relatively new field, LM requires the structured development of specific knowledge, skills, and attitudes during postgraduate training [14]. For LM to be incorporated effectively into medical education, it is first necessary to identify its core learning objectives an effort for which the E-Delphi technique offers a systematic approach [15]. Learning objectives refer to specific and measurable statements of expected learner outcomes. Compared to broader competency frameworks, they provide more actionable elements for curriculum design and assessment.

This study aimed to conduct a needs assessment using the E-Delphi method to define the essential learning objectives for integrating LM into the Family Medicine Residency Training Program.

## Methods

### Study design

This study was designed as methodological research with the objective of identifying the learning objectives for a LM curriculum within the Family Medicine Residency Training Program. An E-Delphi format was utilized, enabling the classical Delphi process to be conducted via digital platforms [16].

The study protocol was guided by established best practices from Delphi applications in the social and health sciences and adhered to the DELPHISTAR guidelines, which provide standardized reporting criteria for interdisciplinary Delphi studies [17].

**Fig. 1:**
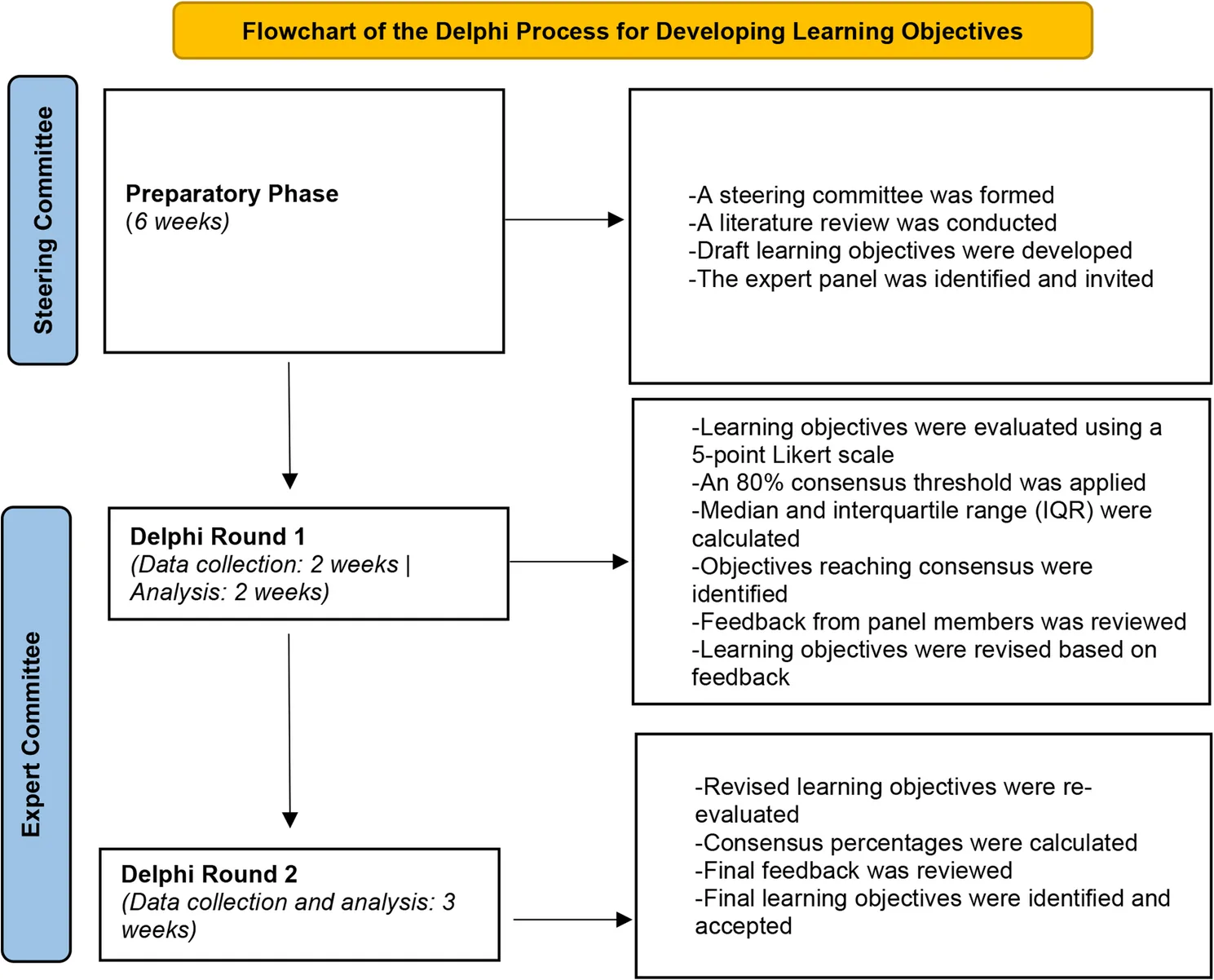
Flowchart of the Delphi process for developing learning objectives. The figure presents the preparatory phase and two Delphi rounds. Learning objectives were evaluated using a 5-point Likert scale, and consensus was defined as ≥ 80% agreement (median ≥ 4, IQR ≤ 1)

The study design is summarized in Fig. 1.

### Study steering group

During the preparatory phase, a Study Steering Group was established, comprising three family medicine academicians, one practicing family medicine specialist, and one family medicine resident. The group brought together experts with extensive clinical and methodological experience in family medicine.

Among the academicians, one held the title of professor and two were associate professors, each affiliated with different university departments of family medicine. All had substantial experience in the design and implementation of medical and family medicine education programs. Notably, one of the associate professors also held a doctoral degree in Curriculum and Instruction, contributing additional expertise in educational design and pedagogy.

The family medicine specialist was in the second year of practice following residency, providing valuable insights into both the current educational structure and post-specialization professional needs. The resident member was in the final year of training, contributing to the relevance of the proposed learning objectives from a trainee perspective.

The Steering Group convened at key stages of the study to design the research protocol, develop and refine the Delphi questionnaire, ensure item clarity and validity, and determine expert panel selection criteria.

### Sample – expert panel selection

There is no universally accepted standard for determining Delphi study sample size; the number of participants typically depends on the study’s purpose, methodological design, and data collection period [18, 19].

Existing literature reports panel sizes ranging from 10 to 1000 participants, although most studies include between 10 and 100 experts. Panels exceeding 100 participants are uncommon due to logistical and analytical challenges [20]. In medical education research, previous Delphi studies defining competencies have generally achieved consensus with 8–18 experts in the final round [21–24].

For this study, experts were defined as professionals with academic or clinical experience in Lifestyle Medicine or its related domains (e.g., nutrition, physical activity, behavioral change, or preventive medicine). Relevant professional experience was defined as prior involvement in clinical practice, teaching, or research related to these areas. Experts were included if they agreed to participate in all Delphi rounds and met at least one of the following criteria: affiliation with family medicine (academic, specialist, or resident), active work in primary care, or professional experience in medical education, or lifestyle medicine related domains. Those unable to complete all Delphi rounds were excluded.

### Sampling method

To ensure a diversity of professional viewpoints, expert panel members were recruited using purposive and snowball sampling strategies. The target panel size was 16 participants.

Purposive sampling identified individuals from key disciplines—family medicine academia, primary care, medical education, dietetics, and sports medicine. Invitations were distributed via email and WhatsApp to relevant academic and professional organizations to ensure multidisciplinary representation.

Snowball sampling further expanded recruitment: initial invitees were encouraged to share the study information with other eligible professionals. This approach enabled inclusion of experts from complementary domains such as nutrition and physical activity, enhancing the interdisciplinary scope of the panel.

Recruitment continued for four weeks, leveraging networks and associations (e.g., Family Physicians Association, Dietitians Association, Sports Medicine Association). The target number of participants (*N* = 16) was achieved within this period. All participants received detailed study information and provided informed consent prior to enrolment. Informed consent was obtained electronically from all participants prior to their participation, with participants indicating their consent before accessing the Delphi questionnaire.

### Procedure

This study employed a two-round electronic Delphi (E-Delphi) process.

#### Preparatory phase – development of initial learning objectives

To define competencies for the targeted educational program, the Steering Group conducted a document analysis and literature review of existing educational frameworks. This process was informed by a review of the international literature (PubMed, Scopus, Web of Science), and the objectives were organized under six core thematic areas. Each proposed objective was discussed by the group; overlapping or inappropriate items were revised or removed through consensus. The resulting list constituted the initial draft of learning objectives. The learning objectives were categorized under the domains of knowledge, attitudes, and behaviours a commonly used framework in competency-based curriculum development [25].

#### Round 1 – initial evaluation of learning objectives

In the first Delphi round, the draft list was distributed electronically via an online survey platform (Google Forms) to the expert panel. Panellists rated the relevance and importance of each objective using a 5-point Likert scale (1 = strongly disagree to 5 = strongly agree), while written comments or suggestions were optional.

Data collection occurred between March and May 2024. Each round remained open for 12 days, with automated reminders sent on Days 8 and 10 and an SMS reminder on Day 10 for non-respondents. A minimum 70% response rate was targeted, consistent with Delphi study recommendations [26].

Round 1 was completed within four weeks. A total of 15 participants completed this round. No additional discussions were conducted between rounds. Feedback was collected exclusively through the online questionnaire, where participants could provide comments via open-text fields. Learning objectives were reviewed by the research team based on feedback.

An item reached consensus if ≥ 80% of panellists rated it 4 or 5, the median was ≥ 4, and the IQR ≤ 1. These thresholds ensured methodological rigor [26]. Comments were reviewed independently. After each Delphi round, the research team systematically evaluated both the quantitative ratings and qualitative feedback provided by the panellists. Written comments were analyzed using a content-based approach. All items were discussed within the research team, and decisions were made by consensus.

Based on this evaluation, items were either retained or excluded if they did not meet the predefined criteria. Objectives with ≥ 80% consensus were accepted, while those below this threshold were excluded. Items that did not reach consensus were removed between rounds. No substantial revision suggestions were provided by the panel members for the remaining items. Therefore, the Delphi process progressed through iterative re-rating of the retained learning objectives rather than content modification. The remaining learning objectives were retained without modification and re-evaluated in subsequent rounds.

In Round 2, the objectives were re-evaluated by the same panel to confirm final consensus. In the second round, the second-draft objectives were redistributed via email to panellists who completed Round 1. Items previously accepted without comment were presented for reference only. The same 80% consensus threshold applied.

Participants received feedback summaries from Round 1 (median, Q1, Q3, IQR) to encourage reflection before confirming their final judgments. Participants were able to provide additional comments or justifications through open-text fields embedded within the questionnaire. For each learning objective, a designated comment box was provided, enabling panellists to elaborate on their ratings, suggest modifications, or provide rationale for their responses after reviewing the feedback summary from the previous round.

### Data analysis

Quantitative survey data were analyzed using descriptive statistics (mean, standard deviation, median, and percentages). Consensus was primarily defined as ≥ 80% agreement among panellists. Median and interquartile range (IQR) values were reported as supplementary descriptive statistics [26].

### Ethics

Ethical approval was granted by the Clinical Research Ethics Committee of Süleyman Demirel University Faculty of Medicine (Decision No: 14/206; Date: 31.10.2023). Prior to participation, all panel members provided informed consent. Each round of the Delphi process was administered electronically and anonymously through Google Forms to ensure accessibility, confidentiality, and consistent data collection.

## Results

In the preparatory phase of the study, the steering committee reviewed the literature and identified a set of learning objectives. A survey form including 114 learning objectives grouped under eight main themes was developed: healthy nutrition, physical activity, sleep health, stress management, avoidance of risky substances, sexual health, healthy social relationships and environmental exposures.

Subsequently, an expert panel was formed. In the first round, 15 participants were reached, including 9 family medicine academicians, 1 family medicine specialist, 1 general practitioner, 1 family medicine resident, 1 medical education academician, 1 academician from the department of nutrition and dietetics, and 1 academician from the department of sports medicine.

The average age of the expert panel members was 44.8 ± 10.7 years. Of the participants, 66.6% (*n* = 10) were women, and 73.3% held a doctoral degree. Their average duration of professional experience was 20.2 ± 10.3 years, ranging from 4 to 41 years. Regarding experience in curriculum development, 73.4% of participants reported feeling partially or fully competent. In terms of duration, 93.3% had at least 1–5 years of experience in curriculum development. When asked about their competence in LM, 66.7% rated themselves as partially, sufficiently, or highly competent. Similarly, 66.7% had 1–5 years or more of experience in working with LM topics (Table 1) .

**Table 1:** Sociodemographic characteristics of expert panel members

Characteristic	% (*n*)
Gender	
Female	66.6% (10)
Male	33.3% (5)
Age (mean ± SD)	44.8 ± 10.7 years (min 28, max 65)
Institution Type	
Tertiary Care	86.6% (13)
Secondary Care	6.6% (1)
Primary Care	6.6% (1)
Position	
Academic	86.6% (13)
Specialist Physician	6.6% (1)
Resident	6.6% (1)
Years in Profession (mean ± SD)	20.2 ± 10.3 years (min 4, max 41)
Curriculum Development Experience	
Sufficient	46.6% (7)
Partially Sufficient	26.6% (4)
Slightly Sufficient	20.0% (3)
Insufficient	6.6% (1)
Mean Experience year in Curriculum Development (mean ± SD)	8.38 ± 7.12 years (min 2, max 20)
LM Experience	
Very Sufficient	6.6% (1)
Sufficient	20.0% (3)
Partially Sufficient	40.0% (6)
Slightly Sufficient	20.0% (3)
LM Experience - Insufficient	6.6% (1)
Years of LM Experience (mean ± SD)	8.5 ± 6.48 years (min 1, max 24)

As a result of the expert panel’s evaluation, consensus was reached on 107 out of 114 learning objectives in the first round. In the second round, 96 of those 107 objectives achieved consensus (Table 2).

**Table 2:** Learning objectives reaching consensus in the second round by the expert panel

Topic	No	Learning Objective	Learning Domain	Consensus Level
				2. Round %
Introduction to Lifestyle Medicine	1	Defines Lifestyle Medicine.	K	100.0
	2	Explains the importance of Lifestyle Medicine.	K	100.0
	3	Describes the relationship between health and lifestyle.	K	100.0
	4	Lists the key components of lifestyle.	K	91.7
	5	Lists effective communication techniques.	K	100.0
	6	Summarizes behavior change methods.	K	91.7
	7	Follows current recommendations related to Lifestyle Medicine.	A	100.0
healthy nutrition	8	Defines healthy nutrition.	K	100.0
	9	Explains the effects of healthy nutrition on general health.	K	100.0
	10	Identifies macronutrients and micronutrients (e.g., carbohydrates, proteins, fats, vitamins, and minerals) and their roles in health.	K	100.0
	11	Describes the health impacts of various vitamin and mineral deficiencies and how they can be addressed through nutrition.	K	100.0
	12	Develops awareness and sensitivity regarding the importance of healthy nutrition.	A	91.7
	13	Evaluates the critical role of nutrition in the prevention and management of diseases.	A	100.0
	14	Develops awareness and attitudes regarding food and nutrition safety principles.	A	91.7
	15	Creates personalized healthy nutrition plans for patients.	B	83.3
	16	Implements disease-preventive nutritional strategies for conditions such as obesity. diabetes. cardiovascular diseases. and cancer.	B	83.3
	17	Provides age-specific nutrition recommendations for different life stages (infancy. childhood. pregnancy. older age. etc.).	B	100.0
physical activity	18	States the importance of physical activity in preventing chronic diseases.	K	100.0
	19	States the importance of physical activity in managing chronic diseases.	K	100.0
	20	Lists the physiological and psychological benefits of regular physical activity for overall health.	K	100.0
	21	Encourages patients to develop positive attitudes toward physical activity.	A	100.0
	22	Identifies barriers to promoting physical activity in clinical practice.	A	100.0
	23	Demonstrates willingness to follow the latest research and guidelines in the field of physical activity and lifestyle medicine.	A	100.0
	24	Integrates individualized physical activity plans into patients’ comprehensive care plans.	B	83.3
	25	Encourages patients to adopt and sustain an active and healthy lifestyle in the long term.	B	100.0
	26	Integrates physical activity and exercise recommendations into patients’ daily routines.	B	91.7
	27	Provides age-specific physical activity recommendations for different life stages (infancy, childhood, pregnancy, older age, etc.).	B	91.7
sleep health.	28	Defines the physiology and structural organization of sleep.	K	100.0
	29	Defines sleep hygiene.	K	100.0
	30	Explains the impact of chronic diseases on sleep patterns and how to manage sleep in such cases.	K	91.7
	31	Identifies at least two lifestyle adjustments (e.g., light exposure, meal timing/composition) that can enhance sleep quality.	K	83.3
	32	Lists ways to improve sleep quality.	K	91.7
	33	Describes lifestyle-based behaviors (activity, nutrition, environment, coping strategies) that can support sleep health.	K	100.0
	34	Develops empathy and understanding toward individuals with sleep disorders.	T	100.0
	35	Recommends lifestyle changes to improve patients’ sleep health.	B	100.0
	36	Supports patients in implementing recommended lifestyle changes to improve sleep health.	B	91.7
	37	Provides recommendations on sleep regulation in individuals with chronic diseases.	B	91.7
Stres management	38	Lists signs of stress (physiological. emotional. and cognitive).	K	100.0
	39	Identifies personal. occupational. and environmental sources of stress.	K	100.0
	40	Explains the negative effects of stress on health.	K	100.0
	41	Explains methods that help reduce the adverse health effects of stress.	K	100.0
	42	Explains the importance and ways of building a personal support network.	K	83.3
	43	Explains motivation strategies to increase job satisfaction.	K	83.3
	44	Lists stress coping strategies in crisis and emergency situations.	K	91.7
	45	Explains the importance of self-care and setting aside regular personal time.	K	83.3
	46	Develops effective communication skills in patient-physician interactions.	A	91.7
	47	Understands the concepts of compassion and emotional regulation.	A	83.3
	48	Recognizes the importance of support networks and evaluates ways to establish them.	A	83.3
	49	Learns. applies. and practices various strategies to reduce and manage stress.	B	91.7
	50	Applies motivation strategies to increase job satisfaction.	B	83.3
	51	Develops and maintains personal self-care habits.	B	83.3
Avoidance of Risky Substances	52	Defines risky substances.	K	100.0
	53	Explains the core concepts of tobacco. alcohol. and substance dependence and their effects on the body.	K	100.0
	54	Describes the characteristics. reasons for use, and health impacts of various addictive substances.	K	91.7
	55	Explains the psychological and social impacts of addiction on individuals’ lives.	K	100.0
	56	Develops empathy and understanding toward individuals with addiction.	A	100.0
	57	Addresses common misconceptions and stigma surrounding addiction and promotes informed attitudes.	A	91.7
	58	Supports effective. evidence-based approaches to addiction treatment.	A	83.3
	59	Assesses addiction-related conditions.	B	91.7
	60	Develops personalized treatment plans for individuals with addiction.	B	83.3
	61	Implements health education and counseling strategies to prevent and reduce tobacco use. alcohol. and substance use.	B	91.7
	62	Communicates effectively and supportively with individuals with addiction.	B	100.0
	63	Refers individuals with addiction to appropriate treatment services.	B	100.0
Sexual health	64	Explains the basic concepts of sexual health and sexuality.	K	91.7
	65	Explains the physiological and psychological aspects of sexual health and sexuality.	K	91.7
	66	Describes fundamental knowledge about fertility. influencing factors. and its relationship with health.	K	91.7
	67	Identifies sexual health problems and disorders and explains their causes. diagnosis. and treatment methods.	K	83.3
	68	Lists sexually transmitted infections (STIs).	K	100.0
	69	Explains methods of protection against STIs.	K	100.0
	70	Explains treatment methods for STIs.	K	100.0
	71	Develops a patient-centered. empathetic. and a non-judgmental attitude regarding sexual health and sexuality.	A	100.0
	72	Builds competence in providing education and counseling on sexual health.	A	83.3
	73	Demonstrates respect for social and cultural differences regarding sexuality and fertility.	A	100.0
	74	Performs sexual health assessments for patients.	B	91.7
	75	Encourages lifestyle changes to improve sexual health and fertility.	B	83.3
	76	Provides patient education on lifestyle changes that improve sexual health and fertility.	B	83.3
	77	Approaches sexual health and fertility from a holistic health perspective.	B	91.7
	78	Coordinates sexual and reproductive health with a multidisciplinary. holistic approach.	B	91.7
healthy social relationships	79	Defines healthy social relationships.	K	100.0
	80	Explains common dynamics and patterns in different types of relationships (romantic, family, workplace, etc.).	K	91.7
	81	Lists qualities of good communication.	K	91.7
	82	Explains the importance of effective communication skills in maintaining healthy relationships.	K	100.0
	83	Develops empathy and understanding in various types of relationships.	A	91.7
	84	Recognizes the importance of personal values and boundaries and raises awareness in this area.	A	83.3
	85	Evaluates the psychological and physical health impact of healthy relationships.	A	91.7
	86	Identifies relationship problems.	B	83.3
environmental exposures	87	Defines environmental health.	K	100.0
	88	Explains the impact of environmental exposures on human health.	K	100.0
	89	Identifies key environmental lifestyle factors (air and water quality, toxin exposure, diet., physical activity, stress management) influencing the development of non-communicable diseases.	K	100.0
	90	Explains the relationship between health problems and environmental exposures.	K	100.0
	91	Explains the biological and physiological mechanisms by which environmental lifestyle factors influence risk and severity of non-communicable diseases.	K	91.7
	92	Assesses individual and community-level risks posed by environmental lifestyle factors.	K	83.3
	93	Explains how to integrate lifestyle medicine with environmental health.	K	100.0
	94	Develops awareness and sensitivity regarding the importance of addressing environmental lifestyle factors in the prevention and management of non-communicable diseases.	A	83.3
	95	Develops an understanding of ethical and equity issues in environmental health.	A	83.3
	96	Participates in public health initiatives that focus on improving environmental lifestyle factors and lifestyle choices to reduce non-communicable disease risk.	B	100.0

*K* knowledge, *A* Attitude, *B* Behavior

In the second round of the study, participants were asked to recomplete the survey by considering the data presented in Round 1. The primary aim of this round was to allow each expert to review the collective response trends, such as group medians and reassess their individual judgments accordingly. This approach enabled participants to modify their prior responses based on the group perspective.

Based on the revised ratings, statistical values, including the first quartile (Q1), third quartile (Q3) median and interquartile range (IQR) were recalculated. If participants changed their opinions, they were asked to reflect these changes using the 5-point Likert scale. This methodology helped clearly distinguish which learning objectives achieved consensus and which did not during the second round.

The second Delphi round was completed over a four-week period. Of the 15 participants who had completed Round 1, 12 continued in Round 2. The mean age of these participants was 42.6 ± 10.5 years, and 58.5% were female. The expert group included 7 family medicine academicians, 1 family medicine resident. 1 general practitioner. 1 family medicine specialist. 1 dietitian. and 1 sports medicine physician.

In this final round, all 107 learning objectives carried forward from the previous round were re-evaluated. Consensus was not reached on 11 of these items, while the remaining 96 achieved the predetermined consensus criteria. The items that did not reach consensus were distributed across the subthemes of healthy nutrition, sleep health, stress management, sexual health, healthy social relationships and environmental exposures (Table 3).

**Table 3:** Learning objectives that did not reach consensus in round 2

Topic	Learning Objective	Q1	Median	Q3	Consensus Level
Healthy Nutrition	Able to integrate food safety principles into clinical practice.	3.25	4	5	75%
Sleep Health	Able to list the stages of sleep.	3.25	4	4.75	75%
	Able to identify common sleep disorders and their key characteristics.	3.25	4	5	75%
	Able to name tools used to assess and manage sleep.	3.25	4	4	75%
Stress Management	Able to list strategies for managing emotional responses.	3.25	4	5	75%
	Able to list methods for reducing stress.	3.25	4	5	75%
Sexual Health	Able to implement appropriate interventions following sexual health assessments.	3	4	5	66.7%
Healthy Social Relationships	Able to provide counseling and guidance on healthy relationship strategies.	3	4	5	66.6%
	Able to educate patients about the importance of healthy relationships and how to maintain them.	3	4	4.75	58.3%
	Able to describe strategies and techniques for conflict resolution in relationships.	3.25	4	5	75%
Environmental Exposures	Able to effectively educate and counsel patients on modifying environmental lifestyle factors to reduce noncommunicable disease risk.	3.25	4.5	5	75%

A high level of consensus was achieved across a substantial proportion of the learning objectives, particularly those addressing the fundamental concepts and core domains of lifestyle medicine. Strong agreement was consistently observed in the domains of healthy nutrition, physical activity, and sleep health, especially for objectives related to basic definitions, underlying mechanisms, and the role of lifestyle factors in health and disease. In addition, objectives reflecting communication skills, empathy, and patient-centered care approaches demonstrated similarly high levels of agreement across multiple domains, including stress management, addiction, and sexual health. Knowledge-based objectives showed the most consistent consensus, while attitude- and behaviour-oriented objectives were also widely supported, particularly those related to patient education, counselling, and the promotion of lifestyle modification.

## Discussion

This methodological E-Delphi study aimed to identify the essential learning objectives for integrating LM into family medicine residency training. Conducted over two rounds, the Delphi process achieved expert consensus on 96 of the 114 proposed objectives, which were organized under eight thematic domains. In this study, learning objectives were conceptualized as specific and measurable statements describing what learners are expected to know, understand, or be able to do following an educational activity. While competencies represent broader, integrated constructs encompassing knowledge, skills, and attitudes, learning objectives offer more granular and actionable elements that can directly inform curriculum design, teaching strategies, and assessment methods. This distinction is particularly relevant in the context of lifestyle medicine, where the absence of a structured educational framework necessitates clearly defined and operationalizable targets.

These objectives form a foundational framework for LM education, encompassing not only knowledge-based competencies but also those related to attitudes and practical skills. This comprehensive approach supports the development of a curriculum that effectively bridges theoretical concepts with real-world behavioral and clinical applications.

High levels of agreement were observed in domains such as nutrition, physical activity, and tobacco and alcohol use, highlighting these as educational priorities. In contrast, consensus was not achieved for several objectives within the domains of sleep health, stress management, and environmental exposures.

The lack of agreement on age-specific nutritional strategies such as those concerning infancy, childhood, pregnancy, and older adulthood may reflect the cross-cutting nature of nutrition across the lifespan. Rather than belonging to a single clinical domain, nutritional care intersects with multiple specialties and must be tailored to physiological changes, disease risk profiles, and social contexts at different life stages. This makes it difficult to define standardized content within a single training framework. Divergent perspectives on how and to what extent these life stage–specific considerations should be incorporated into family medicine training likely contributed to the observed variation in expert responses. Additionally, inconsistencies in the strength and applicability of age-specific guidelines may have further contributed to this lack of consensus [27, 28]. These findings suggest a need for clearer curricular frameworks and case-based learning examples in these areas.

Stress management in clinical education is often limited to personal stress recognition. However, primary care physicians play a key role in addressing behavioural health needs, and brief, feasible interventions can be effectively delivered within routine clinical encounters [29]. Consequently, objectives such as “regulating emotional responses” or “teaching coping techniques” may have faced varied interpretations regarding their relevance and feasibility in primary care. Factors such as time constraints, patient readiness, motivation and systemic support further affect implementation. The literature emphasizes the importance of standardizing both the content and delivery of stress management education [30, 31]. 

Interpersonal and counselling skills require not only cognitive knowledge but also advanced communication competencies, objectives such as “explaining the impact of social relationships on health” or “providing support during relationship crises” may be influenced more by clinical experience, ethical consideration, and individual practitioner style than by structured clinical knowledge. This could explain the lower consensus. Previous studies advocate for structured, supervised educational programs to help family physicians build counselling capacity in these areas [32]. Integrating such content into training requires deliberate pedagogical planning, incorporating approaches such as case-based learning, experiential and simulation-based training, supervised clinical encounters, and interprofessional education.The lack of consensus on interpersonal and counselling skills may reflect differing views among participants regarding the scope and feasibility of these competencies in routine primary care practice. Factors such as time constraints, variability in training backgrounds and the perception that more advanced counselling roles may fall within the domain of other disciplines may have contributed to this variation. Additionally, underlying attributes such as empathy were not evaluated as separate items in this study.

Objectives related to environmental health, such as “developing policies to improve environmental conditions” and “educating patients on environmental exposures.” may have received limited support due to their public health and policy-level implications, opinions varied on how extensively primary care physicians should engage in systemic environmental advocacy. The literature recommends redesigning environmental health education to develop systems thinking and advocacy skills, rather than limiting it to individual awareness [33, 34] This underscores the need for a more clearly defined framework for incorporating environmental health into medical education.

Although topics such as sleep physiology and sleep hygiene are well known in primary care, consensus was achieved in this study primarily for foundational sleep-related objectives, including sleep physiology, sleep stages, sleep hygiene, and lifestyle-based approaches to improving sleep quality. Objectives related to empathy towards patients with sleep disorders and the impact of chronic diseases on sleep were also well supported. However, more practice-oriented competencies such as identifying sleep hygiene components, using sleep assessment and management tools, and recognizing common sleep disorders in detail did not reach consensus across rounds. These objectives may have been perceived as more relevant to secondary care. Concerns about the time, expertise, and infrastructure required to implement these tools may also have influenced panel responses. Additional factors may include variability in participants’ familiarity with sleep medicine, uncertainty regarding the boundaries between primary and specialized care, and differences in how panel members interpreted the expected level of competence (knowledge versus application). While literature supports the use of simple screening tools for early detection of sleep disorders in primary care, it also highlights the limited training available in this area [35, 36] These findings suggest that clearer curricular guidance is needed, including explicit learning objectives, practical recommendations on the use of feasible screening tools in primary care, and alignment with appropriate teaching and assessment methods to support implementation in routine clinical practice.

Taken together the objectives that failed to reach consensus highlight important gaps and priorities in LM education. They provide valuable insight into areas that require further development to ensure a well-rounded training experience.

The identified objectives’ practical applicability is crucial for integrating LM into healthcare providers’ everyday workflows. Training programs must be designed to prepare graduates for the real-life implementation of LM competencies.

Future research should build upon this initial framework by involving larger and more diverse panels and employing alternative methodologies. Additionally, implementation studies assessing the real-world adoption and evaluation of these objectives will be essential for measuring their effectiveness.

This study contributes significantly to the definition of LM competencies in the context of family medicine specialty training. The broad participation and inclusion of multiple disciplinary perspectives helped generate a comprehensive and contextually relevant set of learning objectives.

Although LM is a relatively new discipline its foundational principles particularly the health impacts of lifestyle behaviours have long been acknowledged in medical education. However systematic and structured integration of LM into postgraduate training is a recent advancement. A review of the literature indicates that countries such as the United States and several in Europe have already begun incorporating LM learning objectives into family medicine training programs [37, 38].

In this regard the learning objectives developed in this study can serve as a reference not only for departments of family medicine in Turkey but also for institutions worldwide seeking to embed LM into residency curricula. Given the global relevance of lifestyle-related health challenges these objectives offer a transferable framework adaptable to various medical education systems. As such. this study has the potential to inform curriculum development at both national and international levels.

This study has several strengths, including the use of a structured Delphi methodology and the inclusion of a multidisciplinary expert panel, which enhanced the comprehensiveness and validity of the findings. Future efforts should focus on translating these learning objectives into practical curriculum components, with particular attention to behavioural objectives, which may require more structured teaching strategies, experiential learning approaches, and appropriate assessment methods to support their implementation in clinical practice.

### Limitations

Limitations include potential selection bias, as most panellists were affiliated with academic institutions, which may reduce generalizability to frontline primary care settings. The online format limited dynamic interactions possible in face-to-face discussions, and the applicability of the identified objectives in real educational settings was not assessed. One limitation of this study is the relatively limited representation of experts with a specific background in nutrition, with only one panel member from this field. This may have influenced the depth of domain-specific input. Another limitation of this study is that only a small proportion of participants were actively working in primary care. However, the panel also included family medicine specialists working in secondary and tertiary care settings, reflecting a broader perspective within the discipline.

## Conclusion

This study identified 96 essential learning objectives for integrating Lifestyle Medicine into family medicine residency training through a systematic E-Delphi process. These objectives encompass key domains such as nutrition, physical activity, stress management, sleep health, social relationships, and environmental exposures. Strong consensus in areas like nutrition and physical activity underscores their educational priority, while lower agreement in domains such as stress management and environmental exposures indicates a need for clearer frameworks. Overall, the findings provide a foundation for LM curriculum development and represent one of the first structured needs assessments of its kind in Turkey.

## Data Availability

The data supporting the findings of this study are available upon reasonable request from the corresponding author. Due to ethical considerations and participant confidentiality, data cannot be shared publicly.
